# Relationship between Spectral-Domain Optical Coherence Tomography and Standard Automated Perimetry in Healthy and Glaucoma Patients

**DOI:** 10.1155/2014/514948

**Published:** 2014-06-16

**Authors:** Beatriz Abadia, Antonio Ferreras, Pilar Calvo, Mirian Ara, Blanca Ferrandez, Sofia Otin, Paolo Frezzotti, Luis E. Pablo, Michele Figus

**Affiliations:** ^1^Ophthalmology Department, Miguel Servet University Hospital, Aragon Health Sciences Institute, Isabel la Catolica 1-3, 50009 Zaragoza, Spain; ^2^Department of Surgery, Gynecology and Obstetrics, University of Zaragoza, 50009 Zaragoza, Spain; ^3^Department of Ophthalmology, University of Siena, 53100 Siena, Italy; ^4^Department of Neurosciences, University of Pisa, 56126 Pisa, Italy

## Abstract

*Objective*. To evaluate the relationship between spectral-domain optical coherence tomography (OCT) and standard automated perimetry (SAP) in healthy and glaucoma individuals. *Methods*. The sample comprised 338 individuals divided into 2 groups according to intraocular pressure and visual field outcomes. All participants underwent a reliable SAP and imaging of the optic nerve head with the Cirrus OCT. Pearson correlations were calculated between threshold sensitivity values of SAP (converted to linear scale) and OCT parameters. *Results*. Mean age did not differ between the control and glaucoma groups (59.55 ± 9.7 years and 61.05 ± 9.4 years, resp.; *P* = 0.15). Significant differences were found for the threshold sensitivities at each of the 52 points evaluated with SAP (*P* < 0.001) and the peripapillary retinal nerve fiber layer (RNFL) thicknesses, except at 3 and 9 clock-hour positions between both groups. Mild to moderate correlations (ranging between 0.286 and 0.593; *P* < 0.001) were observed between SAP and most OCT parameters in the glaucoma group. The strongest correlations were found between the inferior RNFL thickness and the superior hemifield points. The healthy group showed lower and weaker correlations than the glaucoma group. *Conclusions*. Peripapillary RNFL thickness measured with Cirrus OCT showed mild to moderate correlations with SAP in glaucoma patients.

## 1. Introduction

Several studies have reported the importance of evaluating retinal nerve fiber layer (RNFL) thickness to diagnose and monitor patients with glaucoma. [1–6] In recent years, different instruments have been introduced to quantitatively measure peripapillary RNFL thickness. One of these techniques is spectral-domain optical coherence tomography (OCT), which has enhanced the axial resolution and decreased the scan acquisition times, improving the ability to diagnose glaucoma through objective, quantitative, and reproducible data. [4, 6–10] However, standard automated perimetry (SAP) still remains the standard test to assess glaucoma damage.

Currently, there is no agreement on whether structural or functional tests are the most sensitive to detect early glaucomatous damage. The relationship between structural and functional tests has been previously evaluated using various imaging devices and different types of perimetries [11–22].

The purpose of this study was to investigate the relationship between the retinal sensitivity evaluated with standard automated perimetry (SAP) and the peripapillary RNFL thickness measured with Cirrus OCT in healthy and glaucoma patients.

## 2. Materials and Methods

The study design adhered to the tenets of the Declaration of Helsinki and was approved by the Institutional Review Board (Clinical Research Ethics Committee of Aragon, CEICA).

Normal eyes were consecutively recruited from patients referred for refraction that underwent routine examination without abnormal ocular findings, hospital staff, and relatives of patients. The glaucoma group comprised subjects with primary open-angle glaucoma, pseudoexfoliative glaucoma, and pigmentary glaucoma. Patients with glaucoma were recruited consecutively from an ongoing longitudinal follow-up study at the Miguel Servet University Hospital. When both eyes fulfilled the inclusion criteria, only one eye per subject was randomly selected.

All of them had to meet the following inclusion criteria: best-corrected visual acuity ≥20/30 (Snellen), refractive error less than 5 spherical diopters and 2 diopters of cylinder, clear cornea, transparent ocular media (nuclear color/opalescence, cortical, or posterior subcapsular lens opacity <1) according to the Lens Opacities Classification System III system [23], and open-anterior chamber angle. The exclusion criteria were as follows: previous intraocular surgery, diabetes or other systemic diseases, history of ocular or neurologic disease, or current use of a medication that could affect visual field sensitivity (deferoxamine, chloroquine or hydroxychloroquine, tamoxifen, phenothiazines, or ethambutol). Ocular hypertensive individuals (intraocular pressure [IOP] higher than 20 mmHg and normal SAP) and patients with normal-tension glaucoma (IOP lower than 21 mmHg and abnormal SAP) were also excluded.

Participants underwent full ophthalmologic examination: clinical history, best-corrected visual acuity, biomicroscopy of anterior segment using a slit lamp, gonioscopy, Goldmann applanation tonometry, central corneal ultrasonic pachymetry (OcuScan RxP, Alcon Laboratories Inc, Irvine, CA), and ophthalmoscopy of the posterior segment.

At least 2 reliable SAPs were performed to minimize the learning effect. [24–26] The visual field was evaluated with a Humphrey Field Analyzer, model 750i (Zeiss Humphrey Systems, Dublin, CA) by using the 24-2 SITA standard strategy. Near addition was added to the subject's refractive correction. If fixation losses were higher than 20% or false-positive or false-negative rates were higher than 15%, the test was repeated. The subjects completed the perimetry measurements before undergoing any clinical examination or structural test. Each perimetry was performed on different days to avoid a fatigue effect. Abnormal SAP results were defined as typical glaucomatous defects with a pattern standard deviation (PSD) significantly elevated beyond the 5% level and/or a Glaucoma Hemifield Test outside normal limits.

The sample was divided into 2 groups according to IOP and visual field outcome, regardless of optic disc appearance. The control group had IOP lower than 21 mmHg and normal SAP, while the glaucoma group had IOP greater than 20 mmHg and abnormal SAP results.

Peripapillary RNFL thickness was measured using the Optic Disc Cube 200 × 200 scanning protocol (software version 6.2) of the Cirrus OCT (Carl Zeiss Meditec, Dublin, CA). This protocol scanned a 6 × 6 mm^2^ area centered on the optic disc. Then, a 3.46 mm diameter circular scan, containing 256 A-scans, was automatically targeted around the optic disc to provide the RNFL thicknesses of the four quadrants and at each of the 12 clock-hour positions. Left eyes were converted to a right eye format. All images were obtained by the same experienced technician with a signal/strength ratio greater than 6/10.

All the ophthalmic examinations, perimetry tests, and OCTs were performed within 6 weeks of the subject's date of enrolment into the study.

### 2.1. Statistical Analysis

All statistical analyses were calculated using IBM SPSS (version 22, IBM Corporation, Somers, NY) statistical software. All the variables studied followed a normal distribution as verified with the Kolmogorov-Smirnov test (K-S of 1 sample). Demographics, SAP, and OCT parameters between both groups were compared with the independent *t*-test.

**Figure 1:**
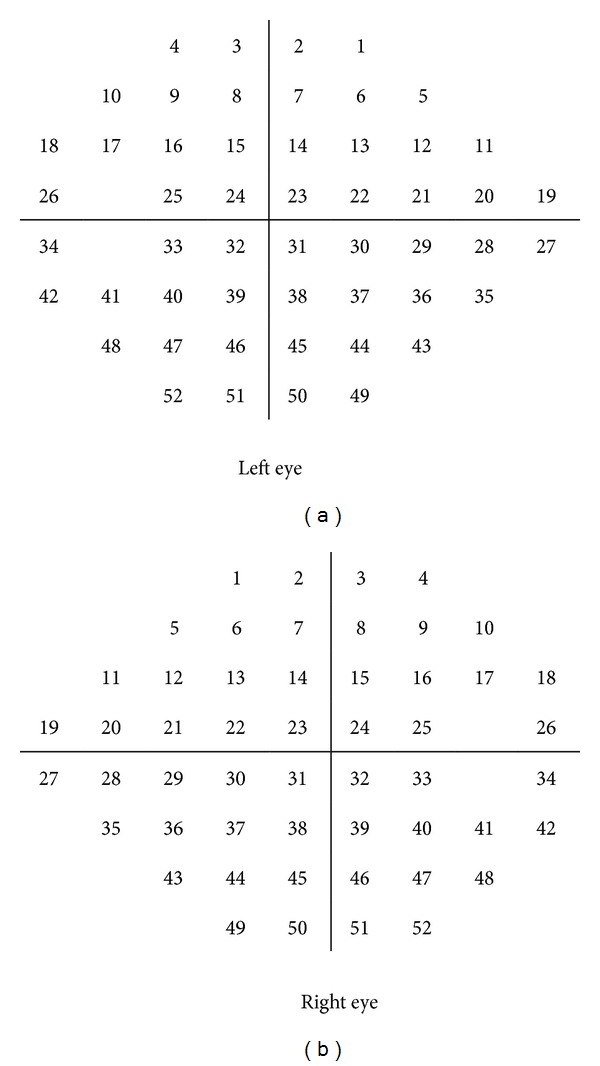
Each of the test points of the 24-2 SITA standard strategy was numbered according to the following grid.

Threshold sensitivity values in decibels (dB) were obtained for each visual field point, numbering from 1 to 26 in the upper hemifield and 27 to 52 in the lower hemifield (Figure 1). These threshold values are tenths of a log unit. The white stimulus presented by the Humphrey perimeter varied in intensity over a range of 5.1 log units (51 dB) between 0.08 and 10,000 apostilbs (asb). 0 dB value corresponded to the maximum brightness that the perimeter can produce (stimulus intensity of 10,000 asb), while 51 dB to the minimum stimulus intensity (0.08 asb). As visual field test points are on a logarithmic scale, the dB levels in each location of the raw numeric plot were converted to a linear scale.

Pearson's correlation coefficients (*r*) were calculated between the RNFL thicknesses measured with OCT and the threshold values of SAP (in a linear scale), according to the anatomic RNFL distribution of the bundles in the retina.

For all analysis, *P* < 0.05 was considered statistically significant. However, when multiple comparisons were performed, the Bonferroni correction was applied to lower the level of significance.

## 3. Results

A total of 350 eyes of 350 subjects were prospectively preenrolled. In 4 cases we could not obtain reliable results in some of the tests and 8 did not complete all the tests included in the study protocol. These 12 subjects were excluded from further analysis. Finally, 338 eyes (182 healthy subjects and 156 glaucoma patients) of Caucasian origin were included in the statistical analysis.

Table 1 shows the clinical characteristics of the study sample. Mean age was 59.55 ± 9.7 years in the control group and 61.05 ± 9.4 years in the glaucoma group (*P* = 0.15). The control group comprised 125 women (68.7%), while the glaucoma group included 88 (56.4%; Chi-square test *P* < 0.001). There were significant differences in baseline IOP, best-corrected visual acuity, central corneal thickness, mean deviation of SAP, PSD of SAP, and visual field index (VFI) of SAP between both groups (*P* < 0.001).

Threshold values at each of the 52 tested points by SAP were different between healthy and glaucoma patients (*P* < 0.001).

Average RNFL thickness measured with Cirrus OCT was 98.61 ± 8.5 *μ*m in the control group and 74.43 ± 15.1 *μ*m in the glaucoma group (*P* < 0.001). Significant differences (*P* < 0.001) were found for the peripapillary RNFL thicknesses, except at 3 and 9 clock-hour positions, between both groups (Table 2).

The control group did not show significant correlations between the RNFL thicknesses measured with Cirrus OCT and the threshold values of SAP. On the other hand, the glaucoma group showed mild to moderate correlations between the RNFL thickness measured with Cirrus OCT and the retinal sensitivity evaluated with SAP. The strongest correlation was found between the inferior RNFL quadrant and the point 8 of SAP (superior hemifield; *r* = 0.534; *P* < 0.001).

**Figure 2:**
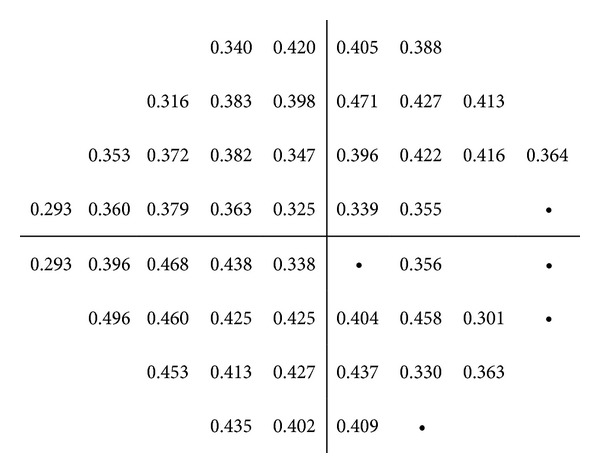
Pearson correlations between the threshold values of SAP (linear scale) and the average RNFL thickness measured with Cirrus OCT, in the glaucoma group. The table only presents the significant correlations (*P* < 0.001).

**Figure 3:**
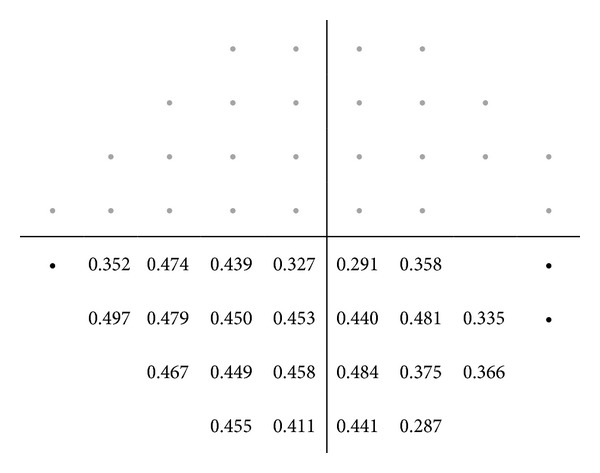
Pearson correlations between the threshold values of SAP (linear scale) and the superior RNFL quadrant thickness measured with Cirrus OCT, in the glaucoma group. The table only presents the significant correlations (*P* < 0.001).

**Figure 4:**
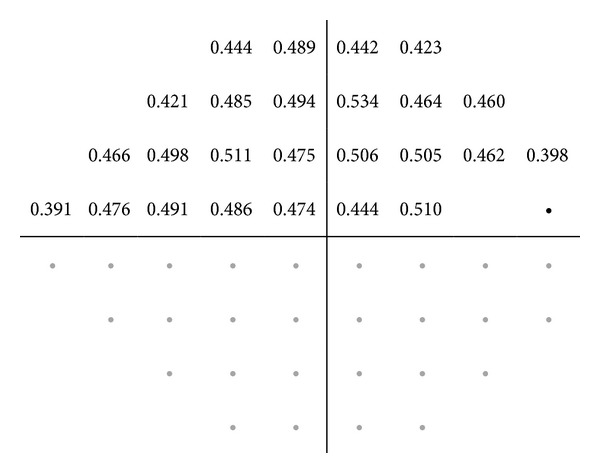
Pearson correlations between the superior threshold values of SAP (linear scale) and the inferior RNFL quadrant thickness measured with Cirrus OCT, in the glaucoma group. The table only presents the significant correlations (*P* < 0.001).

**Figure 5:**
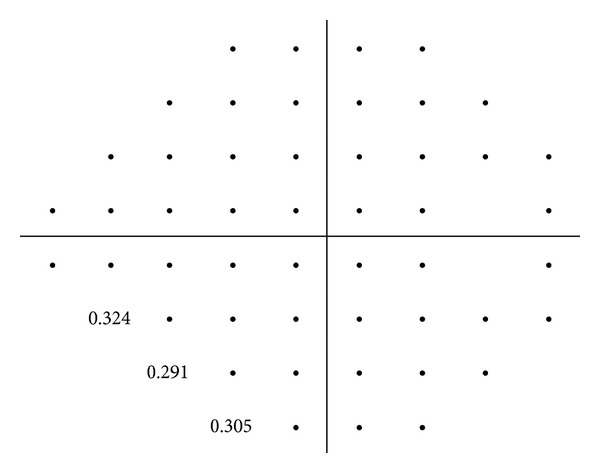
Pearson correlations between the threshold values of SAP (linear scale) and the nasal RNFL quadrant thickness measured with Cirrus OCT, in the glaucoma group. The table only presents the significant correlations (*P* < 0.001).

**Figure 6:**
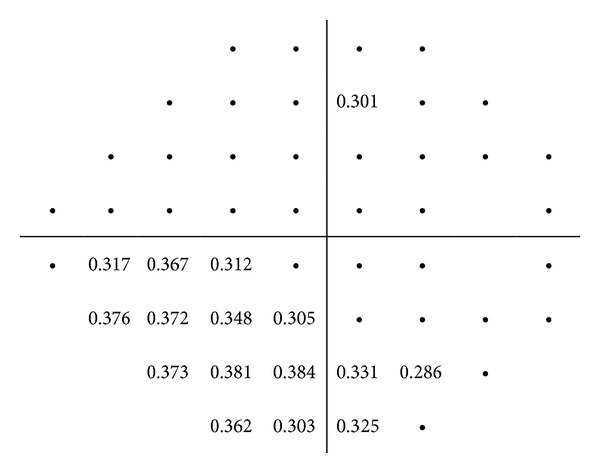
Pearson correlations between the threshold values of SAP (linear scale) and the temporal RNFL quadrant thickness measured with Cirrus OCT, in the glaucoma group. The table only presents the significant correlations (*P* < 0.001).

Figures 2, 3, 4, 5, and 6 show maps of the topographic correlation between both devices. Within the superior hemifield, the strongest correlation was found between point 8 of SAP and the RNFL thickness at 6 clock-hour position (0.485; *P* < 0.001). Within the inferior hemifield, the strongest correlation was found between point 36 of SAP and the RNFL thickness at 11 clock-hour position (0.474; *P* < 0.001).

## 4. Discussion

This study investigated the relationship between Cirrus OCT and the retinal sensitivity measured with SAP in healthy and glaucoma patients. The 52 SAP points, recorded in decibels (logarithmic units), were converted to a linear scale, before calculating the correlations between both instruments, because the RNFL thicknesses measured with OCT were obtained in a linear scale (microns). Nevertheless, because our sample did not include patients with normal-tension glaucoma, our results may not apply to these patients.

Structure-function maps have been previously developed in order to understand the relationship between the optic disc morphology and the corresponding visual field defects [12, 27]. In 2000, Garway-Heath et al. [27] reported one of the most complete structure-function maps in human eyes. Lately, Lamparter et al. [28] described the influence of the variability of normal optic nerve head morphology. The high variability of human RNFL distribution around the optic nerve head and the intertest variability of SAP limit the possibility of obtaining stronger correlations between these tests. Thus, we could not evidence significant correlations between SAP and the peripapillary RNFL thickness in the control group. However, we found mild to moderate correlations in the glaucoma group because the range of change of variables was higher in this group. The RNFL thicknesses or the threshold values of SAP varied widely from mild to advanced glaucoma, and consequently it was easier to determine the correlations in this group than in the control group.

Based on the anatomic distribution of the RNFL bundles in the retina, the superior hemifield corresponds to the inferior RNFL bundles, and the inferior hemifield corresponds to the superior RNFL bundles; thus, the upper threshold values were correlated with the lower peripapillary RNFL thickness, and vice versa. The strongest correlations were found between the RNFL thickness at inferior quadrant and the superior hemifield points. The inferior RNFL thickness showed the greatest thinning (5, 6, and 7 clock-hour positions) and the strongest associations with the superior hemifield in glaucoma patients. Point 8 of HFA (anatomically corresponded with the retinal sensitivity of the arcuate nerve fiber bundle) and inferior quadrant thickness exhibited the strongest correlation (*r* = 0.534; *P* < 0.001). These results were consistent with prior studies and clinical findings, which have been trying to establish the relationship between SAP and OCT. [29–33] El Beltagi et al. [31], in a retrospective study including 43 glaucoma patients, also found that RNFL thicknesses at 6, 7, and 8 clock-hour positions were the thinnest regions in glaucoma patients. The best correlations were observed with the superior arcuate region and nasal step of SAP (range between 0.34 and 0.57). Nilforushan et al. [33] studied the structure-function relationship in 97 patients with suspected or early glaucoma and also reported the highest correlation between the inferotemporal sectors of RNFL and the superonasal visual field (*r* = 0.24).

Some authors suggested that the number of altered points in the superior hemifield is more informative than those located in the inferior hemifield for discriminating between normal subjects and early glaucoma defects. [34, 35] Anton et al. [34] reported that 84% of eyes with focal RNFL defects in the inferior rim corresponded to defects in the superior hemifield.

Our analysis was forced to use the data as these devices provide them. Humphrey perimetry tests visual field points on a grid not arranged according to the anatomy of the nerve fiber bundle paths, while OCT measures the peripapillary RNFL thickness in 12 sectors in which size is independent of the anatomic distribution of the RNFL bundles in the optic nerve head. The mild to moderate correlations found between OCT and SAP in the glaucoma group suggest a reasonable level of relationship in measuring different characteristics of the same disease.

## 5. Conclusions

All peripapillary RNFL thicknesses measured with Cirrus OCT were different between healthy and glaucoma patients, except for the RNFL thickness at 3 and 9 clock-hour positions (horizontal axis). In general, the relationship between the RNFL thickness around the optic disc and the retinal sensitivity evaluated by SAP was moderate in glaucoma patients.

## Figures and Tables

**Table 1:** Clinical characteristics of the study sample.

	Control group		Glaucoma group		P*
	Mean	SD	Mean	SD	
Age (yrs.)	59.55	9.71	61.05	9.43	0.152
BCVA (Snellen)	0.93	0.10	0.82	0.17	<0.001
Baseline IOP (mmHg)	17.10	2.30	26.68	5.61	<0.001
Pachymetry (*µ*m)	554.63	33.74	535.30	37.72	0.001
MD SAP (dB)	−0.38	0.94	−6.64	6.01	<0.001
PSD SAP	1.42	0.25	6.03	3.82	<0.001
VFI SAP	99.55	0.67	83.97	17.17	<0.001
*n*	182		156		

*Student's *t*-test.

BCVA: best-corrected visual acuity; IOP: intraocular pressure; MD: mean deviation; PSD: pattern standard deviation; SAP: standard automated perimetry; VFI: visual field index; SD: standard deviation.

**Table 2:** OCT parameters in the control and glaucoma groups. Differences between groups were calculated by Student's *t*-test (*P* < 0.003 was considered statistically significant).

	Control group				Glaucoma group				*P*
	Min	Max	Mean	SD	Min	Max	Mean	SD	
H1	47	186	108.54	23.43	33	151	81.59	22.19	<0.001
H2	61	138	95.64	18.10	40	127	76.75	18.05	<0.001
H3	24	95	60.46	9.94	23	93	57.54	11.89	0.023
H4	44	112	69.49	12.84	25	96	60.77	12.34	<0.001
H5	72	175	108.93	20.35	35	138	74.64	21.59	<0.001
H6	84	247	144.86	25.57	41	175	92.84	33.07	<0.001
H7	81	208	142.07	22.24	35	176	88.41	34.40	<0.001
H8	39	126	68.48	13.50	28	120	56.74	17.35	<0.001
H9	37	88	51.73	8.34	25	120	49.63	14.37	0.124
H10	44	123	77.76	11.94	20	112	64.49	19.13	<0.001
H11	90	180	133.71	16.40	33	171	95.73	32.02	<0.001
H12	42	203	121.56	28.27	36	148	89.50	25.00	<0.001
Superior	67	175	121.37	17.10	49	148	89.08	21.50	<0.001
Inferior	100	180	132.03	14.97	45	137	85.38	25.32	<0.001
Nasal	55	103	75.27	10.83	25	94	64.62	11.86	<0.001
Temporal	50	104	65.92	9.14	25	112	57.10	14.94	<0.001
Average thickness	80	120	98.61	8.50	47	107	74.43	15.17	<0.001

H: RNFL thickness at clock-hour position; Min: minimum; Max: maximum; SD: standard deviation.

## References

[1] Quigley HA, Miller NR, George T (1980). Clinical evaluation of nerve fiber layer atrophy as an indicator of glaucomatous optic nerve damage. *Archives of Ophthalmology*.

[2] Sommer A, Katz J, Quigley HA (1991). Clinically detectable nerve fiber atrophy precedes the onset of glaucomatous field loss. *Archives of Ophthalmology*.

[3] Ferreras A, Pablo LE, Pajarín AB, Larrosa JM, Polo V, Honrubia FM (2008). Logistic regression analysis for early glaucoma diagnosis using optical coherence tomography. *Archives of Ophthalmology*.

[4] Garas A, Vargha P, Holló G (2010). Reproducibility of retinal nerve fiber layer and macular thickness measurement with the RTVue-100 optical coherence tomograph. *Ophthalmology*.

[5] Pablo LE, Ferreras A, Pajarín AB, Fogagnolo P (2010). Diagnostic ability of a linear discriminant function for optic nerve head parameters measured with optical coherence tomography for perimetric glaucoma. *Eye*.

[6] Leung CK-S, Cheung CY-L, Weinreb RN (2009). Retinal nerve fiber layer imaging with spectral-domain optical coherence tomography. A variability and diagnostic performance study. *Ophthalmology*.

[7] Menke MN, Knecht P, Sturm V, Dabov S, Funk J (2008). Reproducibility of nerve fiber layer thickness measurements using 3D fourier-domain OCT. *Investigative Ophthalmology and Visual Science*.

[8] Mwanza JC, Chang RT, Budenz DL (2010). Reproducibility of peripapillary retinal nerve fiber layer thickness and optic nerve head parameters measured with cirrus HD-OCT in glaucomatous eyes. *Investigative Ophthalmology and Visual Science*.

[9] González-García AO, Vizzeri G, Bowd C, Medeiros FA, Zangwill LM, Weinreb RN (2009). Reproducibility of RTVue retinal nerve fiber layer thickness and optic disc measurements and agreement with stratus optical coherence tomography Measurements. *The American Journal of Ophthalmology*.

[10] Kim JS, Ishikawa H, Sung KR (2009). Retinal nerve fibre layer thickness measurement reproducibility improved with spectral domain optical coherence tomography. *British Journal of Ophthalmology*.

[11] López-Peña MJ, Ferreras A, Polo V, Larrosa JM, Honrubia FM (2007). Relationship between standard automated perimetry and HRT, OCT and GDx in normal, ocular hypertesive and glaucomatous subjects. *Archivos de la Sociedad Espanola de Oftalmologia*.

[12] Ferreras A, Pablo LE, Garway-Heath DF, Fogagnolo P, García-Feijoo J (2008). Mapping standard automated perimetry to the peripapillary retinal nerve fiber layer in glaucoma. *Investigative Ophthalmology and Visual Science*.

[13] López-Peña MJ, Ferreras A, Larrosa JM, Polo V, Fogagnolo P, Honrubia FM (2009). Relationship between standard automated perimetry and optic nerve head topography performed with the Heidelberg Retina Tomograph. *Archivos de la Sociedad Española de Oftalmología*.

[14] Aptel F, Sayous R, Fortoul V, Beccat S, Denis P (2010). Structure-function relationships using spectral-domain optical coherence tomography: comparison with scanning laser polarimetry. *The American Journal of Ophthalmology*.

[15] Lee JR, Jeoung JW, Choi J, Choi JY, Park KH, Kim Y-D (2010). Structure-function relationships in normal and glaucomatous eyes determined by time- and spectral-domain optical coherence tomography. *Investigative Ophthalmology and Visual Science*.

[16] Lopez-Peña MJ, Reras A, Larrosa JM, Polo V, Pablo LE (2011). Relationship between standard automated perimetry and retinal nerve fiber layer parameters obtained with optical coherence tomography. *Journal of Glaucoma*.

[17] Takagishi M, Hirooka K, Baba T, Mizote M, Shiraga F (2011). Comparison of retinal nerve fiber layer thickness measurements using time domain and spectral domain optical coherence tomography, and visual field sensitivity. *Journal of Glaucoma*.

[18] Rao HL, Zangwill LM, Weinreb RN, Leite MT, Sample PA, Medeiros FA (2011). Structure-function relationship in glaucoma using spectral-domain optical coherence tomography. *Archives of Ophthalmology*.

[19] Na JH, Kook MS, Lee Y, Baek S (2012). Structure-function relationship of the macular visual field sensitivity and the ganglion cell complex thickness in glaucoma. *Investigative Ophthalmology and Visual Science*.

[20] Leite MT, Zangwill LM, Weinreb RN, Rao HL, Alencar LM, Medeiros FA (2012). Structure-function relationships using the cirrus spectral domain optical coherence tomograph and standard automated perimetry. *Journal of Glaucoma*.

[21] Kanamori A, Nakamura M, Tomioka M, Kawaka Y, Yamada Y, Negi A (2013). Structure-function relationship among three types of spectral-domain optical coherent tomography instruments in measuring parapapillary retinal nerve fibre layer thickness. *Acta Ophthalmologica*.

[22] Park HYL, Park CK (2013). Structure-function relationship and diagnostic value of RNFL area index compared with circumpapillary RNFL thickness by spectral-domain OCT. *Journal of Glaucoma*.

[23] Chylack LT, Wolfe JK, Singer DM (1993). The lens opacities classification system III. *Archives of Ophthalmology*.

[24] Heijl A, Lindgren A, Lindgren G (1989). Test-retest variability in glaucomatous visual fields. *The American Journal of Ophthalmology*.

[25] Gonzalez-Hernandez M, Pablo LE, Armas-Dominguez K, Rodriguez de La Vega R, Ferreras A, Gonzalez de La Rosa M (2009). Structure-function relationship depends on glaucoma severity. *British Journal of Ophthalmology*.

[26] Fogagnolo P, Sangermani C, Oddone F (2011). Long-term perimetric fluctuation in patients with different stages of glaucoma. *British Journal of Ophthalmology*.

[27] Garway-Heath DF, Poinoosawmy D, Fitzke FW, Hitchings RA (2000). Mapping the visual field to the optic disc in normal tension glaucoma eyes. *Ophthalmology*.

[28] Lamparter J, Russell RA, Zhu H (2013). The influence of intersubject variability in ocular anatomical variables on the mapping of retinal locations to the retinal nerve fiber layer and optic nerve head. *Investigative Ophthalmology and Visual Science*.

[29] Williams ZY, Schuman JS, Gamell L (2002). Optical coherence tomography measurement of nerve fiber layer thickness and the likelihood of a visual field defect. *The American Journal of Ophthalmology*.

[30] Mok KH, Lee VW-H, So KF (2003). Retinal nerve fiber layer measurement by optical coherence tomography in glaucoma suspects with short-wavelength perimetry abnormalities. *Journal of Glaucoma*.

[31] El Beltagi TA, Bowd C, Boden C (2003). Retinal nerve fiber layer thickness measured with optical coherence tomography is related to visual function in glaucomatous eyes. *Ophthalmology*.

[32] Güerri N, Polo V, Larrosa JM, Egea C, Ferreras A, Pablo LE (2013). Functional relationship between retinal sensitivity threshold values assessed by standard automated perimetry in glaucoma. *Archivos de la Sociedad Espanola de Oftalmologia*.

[33] Nilforushan N, Nassiri N, Moghimi S (2012). Structure-function relationships between spectral-domain OCT and standard achromatic perimetry. *Investigative Ophthalmology and Visual Science*.

[34] Anton A, Yamagishi N, Zangwill L, Sample PA, Weinreb RN (1998). Mapping structural to functional damage in glaucoma with standard automated perimetry and confocal scanning laser ophthalmoscopy. *The American Journal of Ophthalmology*.

[35] Yamagishi N, Anton A, Sample PA, Zangwill L, Lopez A, Weinreb RN (1997). Mapping structural damage of the optic disk to visual field defect in glaucoma. *The American Journal of Ophthalmology*.

